# State dependence, personality, and plants: light‐foraging decisions in *Mimosa pudica* (L.)

**DOI:** 10.1002/ece3.2340

**Published:** 2016-08-10

**Authors:** Franz W. Simon, Christina N. Hodson, Bernard D. Roitberg

**Affiliations:** ^1^ Department of Ecology and Evolutionary BiologyYale University165 Prospect StreetNew HavenConnecticut06511; ^2^ Department of BiologyUniversity of Victoria3800 Finnerty RoadVictoriaBritish ColumbiaCanadaV8P 5C2; ^3^ Department of Biological SciencesSimon Fraser University8888 University DriveBurnabyBritish ColumbiaCanadaV5A 1S6

**Keywords:** Behavioral ecology, behavioral reaction norm, behavioral syndrome, energetics, optimal foraging theory, phenotypic plasticity, resource foraging

## Abstract

Plants make foraging decisions that are dependent on ecological conditions, such as resource availability and distribution. Despite the field of plant behavioral ecology gaining momentum, ecologists still know little about what factors impact plant behavior, especially light‐foraging behavior. We made use of the behavioral reaction norm approach to investigate light foraging in a plant species that exhibits rapid movement: *Mimosa pudica*. We explored how herbivore avoidance behavior in *M. pudica* (which closes its leaflets temporarily when disturbed) is affected by an individual's energy state and the quality of the current environment and also repeatedly tested the behavior of individuals from two seed sources to determine whether individuals exhibit a “personality” (i.e., behavioral syndrome). We found that when individuals are in a low‐energy state, they adopt a riskier light‐foraging strategy, opening leaflets faster, and not closing leaflets as often in response to a disturbance. However, when plants are in a high‐energy state, they exhibit a plastic light‐foraging strategy dependent on environment quality. Although we found no evidence that individuals exhibit behavioral syndromes, we found that individuals from different seed sources consistently behave differently from each other. Our results suggest that plants are capable of making state‐dependent decisions and that plant decision making is complex, depending on the interplay between internal and external factors.

## Introduction

Behavior is defined as a phenotypically plastic response to a stimulus, which is rapid and generally reversible (Silvertown and Gordon 1989). Plants have been found to express a number of complex behaviors. For instance, plants incorporate information about the presence and relatedness of neighbors, resource availability and distribution, and risk level into their decisions (Rajaniemi and Reynolds 2004; Dudley and File 2007; Cahill et al. 2010; Jensen et al. 2011). Because of this, plant behavior is an emerging field in behavioral ecology.

Plants, like animals, require resources to maintain their energy state, to grow, and to reproduce. Plants meet their metabolic needs primarily through nutrient uptake by the root system and energy uptake through photosynthesis. Photosynthetic rate, and in turn energy and fixed carbon intake, is related to the amount of light a photosynthetic organ is exposed to (Mooney and Ehleringer 1978). As a result, plants have adopted certain behaviors to maximize their energy intake through photosynthesis (Stoll and Schmid 1998). For instance, plants grow toward light (positive phototropism) and change the position of their leaves to track the movement of the sun through the sky (Koller 1990; De Kroon et al. 2009). While we know that plants are able to incorporate information about their environment into decisions, little is known about the complexity driving such decisions.

Optimal foraging theory is a general theory proposing that an organism faces trade‐offs during foraging and that natural selection will select the optimal strategy during foraging that balances risk and reward to maximize fitness (Brown 1988; Houston et al. 1993; Werner and Anholt 1993). For instance, when residing in a poor‐quality environment, individuals often exert a greater foraging effort and expose themselves to greater predation risk to gain resources compared to when located in a high‐quality environment (Olsson et al. 2002). Past experience also determines behavioral expression. For example, if an individual is unable to feed for some time, its energy stores will decline and it may exhibit riskier foraging behavior to avoid starvation (Mangel and Clark 1986; Houston et al. 1988; Lima 1998). While there are no current examples of state‐dependent foraging in plants, plants can prime their defense systems against herbivory based on past experience (Engelberth et al. 2004). Additionally, plants often expend energy (through either root growth, leaf growth, or leaf movement) in order to gain resources and an increased foraging intensity in at least some cases is accompanied by increased risk. Therefore, although optimal foraging theory is not often applied to plants (especially in the context of light foraging), but it may also apply to plant foraging behavior, as plants face similar trade‐offs to animals (McNickle et al. 2009).

While optimal foraging theory posits a single best solution for a given situation, it has become increasingly clear that different individuals may behave differently from each other even under the same circumstances; thus, there is behavioral variation within populations at the individual level (Sih et al. 2004; Dingemanse et al. 2012). Because of this, behavioral syndromes (or consistent individual differences in behavior across different environments and over time) have recently become a focus in the study of animal behavior (Sih et al. 2004). Numerous animal taxa have been found to display behavioral syndromes (Bell et al. 2009; Adriaenssens and Johnsson 2013; Sweeney et al. 2013) and even genetically identical individuals within a species have been found to display behavioral syndromes (Schuett et al. 2011). Behavioral reaction norms are a new tool in behavioral ecology adopted from quantitative genetics (Gomulkiewicz and Kirkpatrick 1992) that allows researchers to incorporate behavioral plasticity due to environmental differences and genetic variation in behavior of individuals within a single framework (Dingemanse and Dochtermann 2013). Thus, one can explore whether individuals vary in their phenotypes and if there are individual–environment interactions allowing us to explain not only individual differences in behavior, but also whether individuals vary in their plasticity.

**Figure 1:**
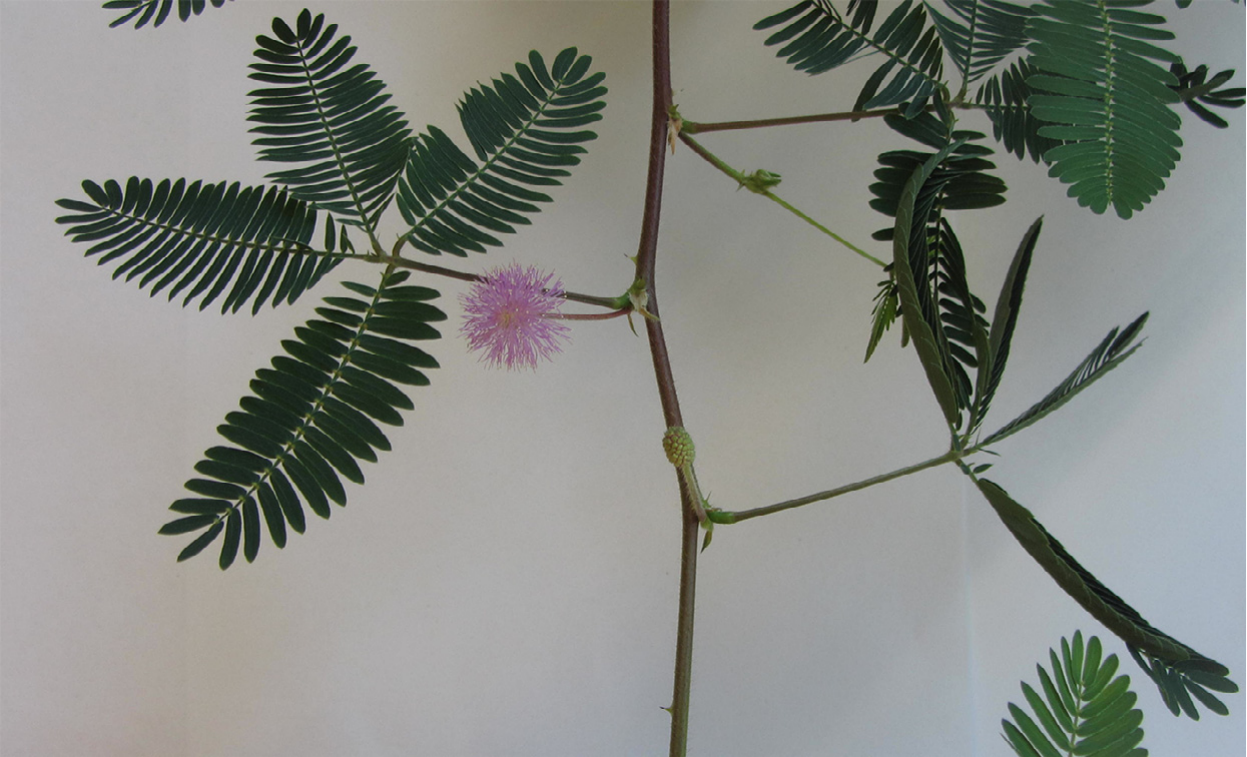
*Mimosa pudica*, the sensitive plant. The image includes a flower, a fully open leaf (left), and a leaf that has been disturbed and has temporarily closed (right). Photographic credit: Christina Hodson.

We investigate how individual *Mimosa pudica* (Linnaeus; the sensitive plant; Fig. 1) make light‐foraging decisions based on several environmental factors. *Mimosa pudica* is an ideal system to work with to investigate behavioral principles in plants as these plants exhibit a rapid herbivore avoidance behavior where they close their leaflets when stimulated and are also able to moderate light‐foraging behavior in response to environmental conditions. *Mimosa pudica* antiherbivore behavior was previously investigated by Jensen et al. (2011) to assess whether plants in darker conditions (i.e., in a resource poor environment) would exhibit riskier behavior and open their leaflets faster after a disturbance compared to plants in light conditions. In congruence with optimal foraging theory, plants were found to take longer to open leaflets after being disturbed when in high resource conditions.

We use a behavioral reaction norm approach to determine how the herbivore avoidance behavior in *M. pudica* changes with current and past light conditions and test whether individuals behave consistently differently from each other (i.e., exhibit a “personality”). We investigate whether *M. pudica* plants make state‐dependent decisions by manipulating the amount of light plants were exposed to 24 h before behavioral assays (causing plants to be either in a low‐energy or high‐energy state) as well as conducting behavioral assays in different light conditions to assess whether individuals exhibit behavioral plasticity depending on environment quality, as Jensen et al. (2011) found. We also repeatedly test *M. pudica*'s herbivore avoidance response over time to investigate whether there is evidence for behavioral syndromes/personalities in this species.

We make several predictions about how *M. pudica* individuals will behave in response to a disturbance. We expect that (1) *M. pudica* plants will display riskier foraging tendencies when in a lower quality environment (Jensen et al. 2011); (2) *M. pudica* individuals will exhibit state‐dependent foraging wherein individuals forage more intensely when energy levels are lower; and (3) like animals, *M. pudica* individuals will exhibit a “personality” such that they will behave consistently relative to other individuals across different contexts and over time.

## Methods

### Study species and rearing information

*Mimosa pudica* is a short‐lived, pantropical plant species that establishes itself in disturbed soils (Francis 2010). *Mimosa pudica* exhibits an herbivore avoidance behavior in which leaflets temporarily close and the petiole droops in response to physical disturbance. This makes leaves less visible to herbivores and exposes spines on the stem of the plant as a defense (Eisner 1981). However, the surface area that is exposed to sunlight is reduced when leaflets are folded, reducing the photosynthetic rate and creating an opportunity cost (Hoddinott 1977). In addition, *M. pudica* plants also exhibit leaf movement in response to light levels, with leaflets closing at night when photosynthesis is not possible (Burkholder and Pratt 1936). We utilized this herbivore avoidance behavior to assess how *M. pudica* makes decisions in response to current and past light levels. We obtained *M. pudica* seeds from two sources (Stokes Seeds and Page's Seeds); henceforth, referred to as Source 1 and Source 2, respectively. Plants were raised under semi‐natural conditions in a greenhouse at Simon Fraser University. To promote germination, we placed seeds in a warm, dark, humid environment for 48 h before planting. We initially planted seeds in groups of three in 10 cm pots in potting mix (Sunshine mix 1, Sun Gro Horticulture). When plants were approximately 2 weeks old, we transplanted them individually into larger pots (20 cm). We watered plants as needed (approximately three times a week) and fertilized monthly.

### Experimental design

We tested the light‐foraging behavior of 56 plants in a 2 × 2 × 2 factorial design with seed source, resource level, and energy state as factors. We began the behavioral assays when individuals were 6 weeks old and continued this assay for the next 8 weeks until each individual plant had been exposed to each treatment four times (in this interval plants had progressed from having their second set of true leaves to flowering). Each plant was exposed to the full set of four treatments in a random order every 2 weeks with two treatment days each week. Therefore, each individual plant was sampled a total of sixteen times. After all the plant behavioral responses were measured on a treatment day, we randomly moved the plants within the greenhouse to avoid long‐term microhabitats or long‐term interactions between neighboring plants.

We wanted to determine how light‐foraging behavior was affected by past resource levels as well as current light‐foraging conditions. Therefore, we manipulated the amount of light plants were exposed to the day of the behavioral assay (i.e., the current resource level the plant was exposed to) as well as the day prior to the behavioral assay (manipulating the energy state of the plant). We exposed plants to either ambient light conditions or a reduced light condition in which we placed a shade cloth over a frame surrounding the plants, which reduced the light level plants experienced by approximately 90%. We placed plants in the treatment manipulating their energy state 24 h prior to the behavioral assay until 3 h before the assay began (i.e., at the beginning of the day of the behavioral assay), at which point they were exposed to the light conditions they would be in during the assay. We expected that plants in the low light treatment 24 h prior to the behavioral assay would be in a lower energy state as they were deprived of the ability to photosynthesize and had to use their energy reserves for maintenance and growth while plants that were exposed to full light would have been able to continue foraging for light (Pettersson and Brönmark 1993). Behavioral assays took place at approximately midday each treatment day. The observers were blind to the source of the plant, and whether the plants had been deprived of light the previous day. Current light levels were nonblinded due to the same observer measuring the current light levels and performing the behavioral assay.

### Behavioral assay

We measured the temperature in the greenhouse prior to behavioral assays as temperature has been found to affect leaflet folding response (Wallace 1931). For each individual plant, we identified the focal leaflet as one leaflet in the second fully developed leaf from the top of the main axis of the plant. We measured the width of the largest point across the focal leaflet and using a light meter (LI‐250A, LI COR Biosciences, NE) measured the light level (in *μ*mol photons m^−2^ sec^−1^) immediately above the focal leaf. We mechanically disturbed the petiole of the focal leaf (which was meant to simulate a potential herbivore attack) by dropping a 36 g 1.5 cm washer attached to a string held by the experimenter 2 cm above the petiole, so that the washer struck the petiole of the leaf. This ensured each plant was disturbed with the same force. We recorded the width of the focal leaflet again immediately after the disturbance. We then recorded the time it took the focal leaflet to open to 70% of its initial width (consistent with previous studies; Jensen et al. 2011). We calculated the leaflet opening rate by dividing the amount the leaflet opened during the assay (i.e., the difference of the leaf's width when closed and its width when 70% open) by the amount of time it took to reopen. In some cases, leaflets did not close to less than 70% of the initial width after being disturbed. In these cases, we were unable to measure leaflet opening rate; however, we were able to calculate whether a plant showed a significant response to disturbance.

### Data analysis

We investigated the effect of the light treatments on two response variables: The amount of time it took the focal leaflet to reopen to 70% of the initial size (opening rate), and whether a plant closed its leaflet more than 70% after being disturbed (responsiveness). All data analyses were performed in R version 3.0.2 (R Core Team 2013). We analyzed data using the lme4 package (Pinheiro et al. 2014). We excluded observations from the opening rate calculations for plants that did not close their leaflets more than 70% of their initial width while analyses measuring responsiveness of plants included all observations. Using generalized linear mixed‐effects models, we examined the opening rate of leaflets using a normal distribution with an identity link and responsiveness of leaflets using a binomial distribution with a logit link. We included the following variables as fixed effects: seed source, whether the plant had been covered by shade cloth the day before the behavioral assay (energy state), whether the plant was covered during the behavioral assay (environment quality), temperature, and light level at the time of the behavioral assay (light level). As observations from the same individual are not independent, we included individual (ID) as a random effect and current light level as a random slope. We compared several candidate models using an Akaike information criterion (AIC) approach starting with a full model with interaction effects between the fixed effects as well as random intercepts and slopes. We then selected the model that minimized the AIC (Tables 1 and 2 include a subset of top models). We used an AIC approach because we had several alternate hypotheses about how individual *M. pudica* plants might make light‐foraging decisions; an AIC framework allows comparisons of different hypothesis and random effects structures (Zuur et al. 2009). The top model was then refitted using restricted maximum likelihood to estimate parameter effects.

**Table 1:** The subset of models describing *Mimosa pudica* leaflet opening rate after a disturbance. We chose from models that tested for interactions between a plant's energy state and current environment condition while also inspecting for seed source and individual effects. Using AIC values, we found the model that included the seed source, the current light levels, energetic state, and environment quality best explained how fast a plant opened its leaflets after a disturbance

ΔAIC	Competing models
0	Opening rate = seed source + light level + energy state + light level × energy state + ID
12.937	Opening rate = seed source + light level + energy state + light level × energy state + energy state × environment quality + ID + ID × Light
19.930	Opening rate = seed source + light level + energy state + environment quality + light level × energy state + energy state × environment quality + ID
34.344	Opening rate = seed source + light level + energy state + energy state × environment quality + ID
47.577	Opening rate = seed source + light level + energy state + ID
56.545	Opening rate = seed source + ID

**Table 2:** The subset of models describing responsiveness of *Mimosa pudica* (i.e., whether a plant closed its leaflets) after a disturbance. These models incorporate plant responsiveness as a function of current light, energetic state, seed source, and individual identity. Using AIC values, we found the model that included a seed source, current light levels, environment quality, and energetic state to be the best descriptor of whether the focal leaf on a plant closed after being mechanically stimulated

ΔAIC	Competing models
0	Responsiveness = seed source + light level + energy state + environment quality + light level × energy state + energy state × environment quality + ID
0.5795	Responsiveness = seed source + light level + energy state + energy state × environment quality + ID
5.7399	Responsiveness = seed source + light level + energy state + light level × energy state + energy state × environment quality + ID + ID × Light
32.592	Responsiveness = seed source + light level + energy state + ID
36.480	Responsiveness = seed source + light level + energy state + light level × energy state + ID
40.259	Responsiveness = seed source + ID

We also assessed whether individuals displayed a behavioral syndrome in two ways by first using model selection to assess different random effects structures, and also via the intraclass correlation (measuring individual repeatability) of our top model. We included individual as a random intercept, and individual x current light levels as random slopes. If random intercept was maintained, this provides evidence that individuals behaved on average differently from each other, and if the random slope of current light level was maintained in the model, this would suggest that different plants reacted more or less plastically to current light level. Additionally, we calculated the intraclass correlation to assess how repeatable individual behavior was. The intraclass correlation was calculated by fitting the top model with individual as a random intercept. The intraclass correlation in this context is the ratio of the variation within an individual's response to the total variation (the sum of the variation between and within individuals; Nakagawa and Schielzeth 2010). We calculated the intraclass correlation for the entire time interval of the experiment and by subsampling our data over the course of each consecutive 2 weeks. This allowed us to determine whether repeatability was a long‐term phenomenon (i.e., over the entire study period), or an ephemeral pattern, respectively.

## Results

### Response to environment and state

The top model describing leaflet opening rate after a disturbance contained seed source, current light level (light level), whether the plant was previously light‐deprived (energy state), and whether it was covered during the trial (environment quality) as explanatory variables and individual as a random effect. The best linear mixed‐effects model for opening rate was

Openingrate=seedsource+lightlevel+energystate+lightlevel×energystate+ID

**Figure 2:**
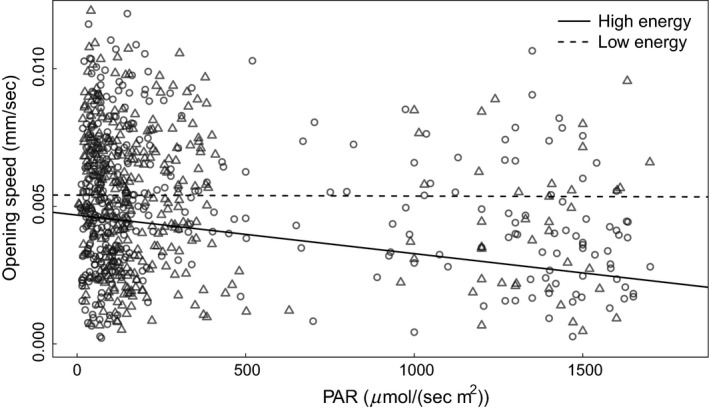
Opening rate of *Mimosa pudica* leaflets in response to light conditions. Regression lines depict mean expected opening rate after a disturbance with respect to current light levels (photosynthetically available radiation) for individuals in high and low‐energy states. Plants that have been deprived of light (low energy) open faster after a disturbance than plants that were not deprived of light (high energy). The raw opening rates of individuals are included on the plot. The circles indicate high‐energy individuals while the triangles indicate individuals in a low‐energy state. All points are transparent to increase the visibility of the data.

**Figure 3:**
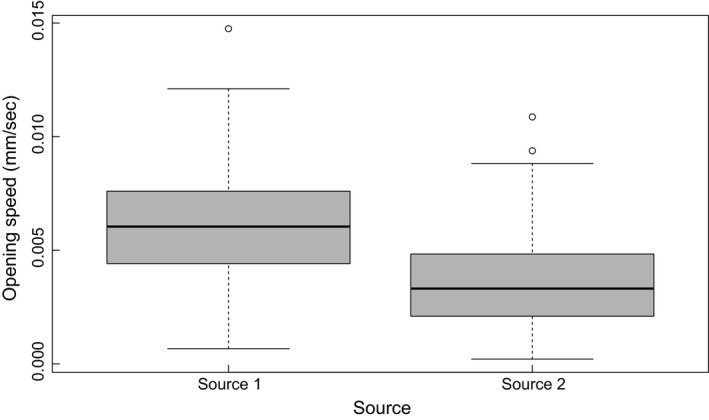
Mean effect plot of the opening rates of plants from Source 1 (*N* = 31) and Source 2 (*N* = 25) after a disturbance. Plants from Source 1 open leaflets faster than Source 2. The solid black line is the median opening speed while the box encloses the 25th‐75th percentiles of data.

The effect sizes, and their *t*‐scores, are reported in Table 3. Additionally, the subset of top models to describe opening rate are in Table 1. When plants were deprived of light, they opened their leaflets faster after a disturbance compared to when they were not previously deprived of light; however, individuals showed no plasticity based on current resource levels when they were light‐deprived. When individuals were in a high‐energy state, they opened their leaflets slower when it was brighter (i.e., resource level was higher) compared to darker conditions (Fig. 2). Additionally, the two plant sources consistently reopened their leaflets at different rates (Fig. 3).

**Table 3:** Parameter values from the best linear mixed effects model of *Mimosa pudica* opening speed after a disturbance. Plants open leaflets faster if they are in a low‐energy state. If plants are in a high‐energy state, they plastically open leaflets depending on light levels (individuals = 56, observations = 721)

Parameter	Effect size	*T*‐score
Intercept	6.32E‐03	33.22
Seed source	−2.42E‐03	−10.05
Light level	−7.94E‐07	−3.88
Low‐energy state	−4.27E‐05	−0.22
Light level × energy state	2.25E‐08	0.06

As measuring opening rate excluded all observations in which individuals did not close their leaflets more than 70% of the original width, we fit a logistic model assessing whether a plant was responsive to a disturbance (i.e., closed its leaflets more than 70% of the initial width). This allowed for inclusion of all data points and allowed us to assess whether some plants were more responsive than others. The top model for describing whether a plant would close its leaflets after being disturbed (responsiveness) included energy state, seed source, light level, environment quality, and interactions between light level and energy state and energy state and environmental quality as explanatory variables and individual as a random effect.

$$\begin{array}{cc} \text{Responsiveness}=\; & \text{seed}\;\text{source}+\text{light}\;\text{level}+\text{energy}\;\text{state}\\ & +\text{environmental}\;\text{quality}\\ & +\text{environmental}\;\text{quality}\times \text{energy}\;\text{state}\\ & +\text{light}\;\text{level}\times \text{energy}\;\text{state}+\text{ID} \end{array}$$

**Figure 4:**
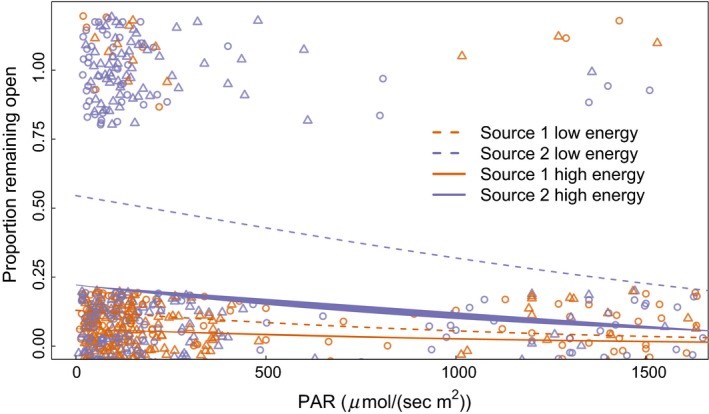
Responsiveness of *Mimosa pudica* leaflets after a disturbance. Whether an individual responded to mechanical stimulation by closing leaflets more than 70% of the initial width is plotted. If the focal leaflet remained open, the value is 1 and if it closed, the value is zero. The raw data are reported in the plot with jitter added on the *y*‐axis to make the distribution of the data visible. Reported are the logistic regression lines based on current photosynthetically available radiation (PAR), energy state, and seed source. Individuals are less likely to close their leaflets after a disturbance if light levels are low. Additionally, individuals were more likely to keep leaflets open when deprived of energy. The raw data are plotted, and the lines are the predicted proportion remaining open from a logistic regression (AIC = 594). Purple points are observations from individuals from Source 1, and the orange points represent observations from individuals from Source 2. The circles represent high‐energy plants, and the triangles represent low‐energy plants.

The parameter values and their t‐scores, are reported in Table 4. Additionally, a subset of the top models describing leaflet responsiveness is found in Table 2. Plants were more likely to close their leaflets if light levels were higher (Fig. 4). If plants were previously deprived of light, they were more likely to keep their leaflets open (Fig. 4). Additionally, plants from different seed sources behaved differently with plants from Source 2 being less likely to close their leaflets compared to plants from Source 1 (Fig. 4). Plants from this source also opened their leaflets faster.

**Table 4:** The parameter values of a logistic regression with individual as a random effect of *Mimosa pudica* responsiveness after a disturbance (AIC = 597, observations = 856, individuals = 56). A negative value indicates plants were more likely to close the focal leaf after the disturbance. Plants in bright conditions were more likely to close leaflets, while plants in a poor‐quality environment were less likely to respond to a disturbance

Parameter	Effect value	*T*‐value
Intercept	−3.67	−8.545
Seed source 2	2.33	8.586
Light level	−0.000423	−0.954
Low‐energy state	1.76	4.084
Low environment quality	0.651	1.675
Light level × low‐energy state	−0.00105	−1.588
Low‐energy state × low environment quality	−2.52	−0.5010

### Behavioral syndrome

Both the top models for leaflet opening rate and responsiveness did not retain the random slope of current light levels in any of the top models. As these models did not retain slope as a random effect, there is little support for the plants having different levels of plasticity. We further quantified how repeatable individual plant behavior was by fitting the top model with individual as a random intercept and taking the intraclass correlation (Nakagawa and Schielzeth 2010). Repeatability was equally low (<10%) across the entire experiment and over 2 week intervals.

## Discussion

Using a behavioral reaction norm approach, we made use of the herbivore avoidance response of *M. pudica* and manipulated environment quality and individual energy state to determine how *M. pudica* makes light‐foraging decisions. We measured whether *M. pudica* plants responded to a disturbance by closing their leaflets and measured how fast they reopened their leaflets after the disturbance. Additionally, by repeatedly testing individual behavior over several weeks, we were able to determine whether *M. pudica* exhibits a personality (i.e., behavioral syndrome).

We found that *M. pudica* will exhibit riskier resource acquisition behavior (i.e., open leaflets faster after a disturbance) when in a low‐quality environment compared to a high‐quality environment, similar to what was found by Jensen et al. (2011). However, we found that this is conditional on the plant being in a high‐energy state, having not been previously deprived of light. When plants are in a low‐energy state, they no longer respond plastically to current light levels and will open their leaflets at a faster rate after a disturbance regardless of current resource levels. This suggests that plants that have previously been deprived of light are energetically constrained and open their leaflets quickly because they need to photosynthesize. This is consistent with optimal foraging theory, which predicts that energetically constrained individuals will take greater risks in order to acquire energy (Mangel and Clark 1986).

Additionally, we found that when plants are in a lower energy state they become less responsive and do not close their leaflets as often after a disturbance. This also supports the interpretation that plants need to forage after being deprived of light and will exhibit riskier behavior in order to gain energy. This is in accordance with theoretical (Ma and Roitberg 2008) and empirical research (Skutelsky 1996), based on a well‐known phenomenon that, as energy reserves decrease, an organism's behavior becomes bolder in order to gain resources. Optimal foraging theory is intended as a general theory, applicable to many taxa. Therefore, these results strengthen optimal foraging theory by showing it is applicable to plant light‐foraging behavior. Additionally, we found that theoretical predictions of state‐dependent foraging also apply to plant behavior, a taxon to which it has not previously been applied.

We found that *M. pudica* incorporates current energy levels and current environment conditions into foraging decisions, but whether foraging decisions are made at the leaf, branch or plant level is still unknown and would be an intriguing area of future research. Plants are modular organisms, made up of distinct units. These units act somewhat autonomously but also coordinate with each other in order to function (De Kroon et al. 2009; McNickle et al. 2009), and it is thought that plant behavior is affected both by local and global (i.e., entire plant) conditions (Schlichting 1986). In addition, it would be interesting to determine how the severity (i.e., length) of light deprivation affects herbivore avoidance response and how this interacts with the locality at which decisions are made. For instance, how does a leaf in a high‐quality (i.e., light rich) environment behave if the rest of the plant is in a poorer quality environment and does the length of time a leaf (or plant) is in a resource poor environment affect behavior? Clearly, there are many aspects of foraging behavior left to explore in both *M. pudica* and other plant species.

Due to our use of the behavioral reaction norm approach, this is the first study to determine how variation in plant behavior is partitioned within individuals, among individuals and between seed sources. We found that the two seed sources we investigated acted differently from each other but that there was no evidence for repeatable individual behavior. We used seeds from two different sources and found that Source 1 consistently opened leaflets faster and was more likely to close its leaflets compared to Source 2. The fact that the two seed sources used in this study exhibited consistently different behavior suggests that a plant's genetic background may influence its behavior. In clonal insects, where within‐clone genetic variation is absent, individuals within the same clonal lineage behave consistently differently from each other (Schuett et al. 2011). Given this, it is intriguing that we were unable to find differences in behavior between individuals from the same seed source in this plant species (especially as in our case, we would not expect individuals from the same source to be genetically identical). However, there is reason to be cautious in interpreting the finding that individuals from the same seed source lack consistent behavioral differences in *M. pudica*. We raised plants in an environment lacking social interaction, and as much as possible removed any environmental variation between individuals during development. We wanted to avoid environmental factors other than the treatments from affecting plant behavior. However, if environmental factors are important in the formation of behavioral consistency in this species, we likely would have been unable to detect individual behavioral syndromes. Our results suggest that the amount of behavioral consistency may be unique to the system under investigation and that in some systems genetics may play a larger role in determining behavioral consistency while in others environment may.

Organisms are more likely to have individual repeated differences if they experience consistent microhabitats and long‐term social interactions (Dingemanse and Wolf 2010). As plants are sessile, they are unable to alter the quality of their environment through movement. Given this, we might expect that the environment would be an important driver of between individual differences in behavior. Additionally, the fact that plants are sessile means that they are locked into long‐term interactions with their neighbors. This means, for instance, that plants that grow faster will be taller than their neighbors, shading their shorter neighbors and perhaps creating a positive feedback loop which could create long‐term differences in behavior between individuals (Luttbeg and Sih 2010). Future studies should address whether rearing environment and long‐term social interactions affect *M. pudica* behavior.

## Conclusion

*Mimosa pudica* exhibits plastic behavior that is determined by both energy state (internal factors) and environment quality (external factors). The presence of state dependence in *M. pudica* opens up new avenues of research into the possibility of state dependence in other plant species and raises more questions about how plants make decisions. The continued application of behavioral ecology to plants has provided increased insight into the principles of plant behavior but also allows for insight into behavioral ecological theory. Studying behavior in plants may be in many ways more tractable than studying behavior in animals as we can control variables (such as social structure) more easily in plant systems than in animal systems due to their sessile nature. Additionally, *M. pudica* is a good model organism for studying how behavior can differ between different hierarchal scales, given that we can study not only individual differences in behavior but variation within different parts of a plant as well. Many principles in ecology were either not formed with any specific taxonomic group in mind or can be applied to other systems than the one that they were designed for. We believe that the continued application of ecological theories to plants not only provides insight into plant behavior but also informs behavioral ecological theory, as demonstrated through this study.

## Data Accessibility

All data used in this manuscript are archived at Dryad. doi:10.5061/dryad.8852k.

## Conflict of Interest

None declared.
